# Methicillin-resistant *Staphylococcus aureus* in Horses and Horse Personnel, 2000–2002

**DOI:** 10.3201/eid1103.040481

**Published:** 2005-03

**Authors:** J.S. Weese, M. Archambault, B.M.Willey, H. Dick, P. Hearn, B.N. Kreiswirth, B. Said-Salim, A. McGeer, Y. Likhoshvay, J.F. Prescott, D.E. Low

**Affiliations:** * University of Guelph, Guelph, Ontario, Canada; † Mount Sinai Hospital, Toronto, Ontario, Canada; ‡ Vita-Tech Laboratories, Markham, Ontario, Canada; § Hearn Veterinary Services, Orangeville, Ontario, Canada; ¶ New York University Medical Center, New York, New York, USA

**Keywords:** methicillin resistance, *Staphylococcus aureus*, cross infection, zoonoses, community-acquired infections, veterinary, horse, research

## Abstract

Methicillin-resistant *Staphylococcus aureus* (MRSA) infection was identified in 2 horses treated at a veterinary hospital in 2000, prompting a study of colonization rates of horses and associated persons. Seventy-nine horses and 27 persons colonized or infected with MRSA were identified from October 2000 to November 2002; most isolations occurred in a 3-month period in 2002. Twenty-seven (34%) of the equine isolates were from the veterinary hospital, while 41 (51%) were from 1 thoroughbred farm in Ontario. Seventeen (63%) of 27 human isolates were from the veterinary hospital, and 8 (30%) were from the thoroughbred farm. Thirteen (16%) horses and 1 (4%) person were clinically infected. Ninety-six percent of equine and 93% of human isolates were subtypes of Canadian epidemic MRSA-5, *spa* type 7 and possessed SCC*mec*IV. All tested isolates from clinical infections were negative for the Panton-Valentine leukocidin genes. Equine MRSA infection may be an important emerging zoonotic and veterinary disease.

## Abstract 2

Methicillin-resistant *Staphylococcus aureus* was isolated from horses and horse personnel in a pattern suggestive of interspecies transmission of a human-origin clone.

Methicillin was first introduced in human medicine in the 1950s for the treatment of penicillin-resistant staphylococci, and within a few years, methicillin-resistant isolates of *Staphylococcus aureus* (MRSA) were identified (*1*). Since then, MRSA has emerged as an important problem in human medicine internationally, especially in the hospital setting (*2*–*6*). Methicillin resistance is mediated by production of an altered penicillin-binding protein (PBP2a), which confers resistance to all β-lactam antimicrobial agents. The gene that encodes this altered PBP, *mecA*, resides on a large, mobile genetic element called the staphylococcal chromosomal cassette *mec* (SCC*mec*). Three types of SCC (types I, II, and III) were originally described in hospital-acquired MRSA strains, most of them isolated before 1990. A fourth type (type IV) was recently described, initially in community-acquired MRSA isolates (*2*–*4*). Although MRSA have been typically hospital acquired (*5*,*6*), reports of community-acquired MRSA in persons have increased (*7*–*9*). In Canada, 6 epidemic clones, designated CMRSA (Canadian epidemic MRSA) 1 through 6 based on pulsed-field gel electrophoresis (PFGE), are recognized (*10*,*11*). In addition to PFGE, SCC*mec* typing and DNA sequencing of the X region of the protein A gene (*spa* typing) can be used to further differentiate strains for epidemiologic analysis (*12*).

The role of MRSA in veterinary medicine has not been well characterized. Isolation of MRSA has been reported in horses, cattle, and dogs (*13*–*20*). In addition, MRSA infection in 2 horses treated at the Ontario Veterinary College Veterinary Teaching Hospital (OVC-VTH) was first documented in 2000. To evaluate the apparent emergence of MRSA infection and colonization in horses, nasal swabs from horses and persons at OVC-VTH and a select group of farms from southern Ontario were cultured.

## Materials and Methods

### Veterinary Hospital Facility

The Large Animal Clinic at OVC-VTH is a tertiary-care referral center with a caseload of ≈2,000 horses per year. Hospital personnel that are in contact with horses include senior clinicians (internists, surgeons, other specialists), residents, interns, veterinary technicians, agricultural assistants, and veterinary students.

### Sampling Procedures

A limited organized MRSA screening of horses at OVC-VTH was performed from October 1 to October 5, 2000, after the first 2 clinical cases were recognized. A more thorough screening program was performed from May 17 to November 16, 2002, after further cases were recognized. With this program, nasal swabs were collected from all horses at the time of admission, weekly during hospitalization, and at the time of discharge. Intermittent screening of horses on 1 Ontario breeding farm (farm A) was performed from May 11 to September 15, 2002, after infected horses were identified. Nasal swabs were collected from horses at 9 other Ontario farms after MRSA infection in a resident horse was identified at OVC-VTH. Nasal swabs were collected from all horses that were present on these farms on the day of sampling.

Voluntary screening of OVC-VTH Large Animal Clinic personnel was performed on 2 occasions in 2002 in response to identification of clusters of nosocomial MRSA colonization in horses. Periodic nasal cultures of horses and personnel were initiated at farm A from May 11 to September 15, 2002.

For persons, cotton-tipped swabs were used to sample both anterior nares. For horses, the swab was inserted ≈10 cm into 1 anterior nare and rubbed against the mucosa as the swab was removed. Swabs were placed in liquid Stuart’s or Amies medium and stored at 4°C until processing. Colonization or infection identified within 72 h of admission was classified as community acquired.

### MRSA Identification, Characterization, and Typing

Nasal swab samples were injected onto mannitol-salt agar with 2 μg/mL oxacillin and incubated aerobically at 35°C for 48 h. Colonies were identified as *S. aureus* based on colony morphology, Gram stain appearance, ability to ferment maltose, and positive tube coagulase test or latex agglutination test (Pastorex Staph Plus, Bio-Rad Laboratories Ltd, Mississauga, Canada).

Screening for methicillin resistance in all *S. aureus* was by growth on Mueller-Hinton agar with 4% NaCl and 6 μg/mL oxacillin. Confirmation of methicillin resistance was by detection of PBP 2a by using the MRSA SCREEN antibody kit (Denka Seiken Co. Ltd, Tokyo, Japan) (*21*).

Antimicrobial susceptibility testing was performed by broth microdilution as per NCCLS guidelines (*22*). Detection of inducible *erm* gene-mediated clindamycin-resistance (MLS_B_ phenotype) was performed by using the double disk diffusion method as described in M100-S14 NCCLS 2004 Informational Supplement (*23*).

Isolates were typed by using PFGE after DNA extraction and *Sma*I digestion (*24*). PFGE images were read manually by 1 investigator (B.W.), and related isolates were divided into subtypes by using an arbitrary naming system based on previously described principles (*25*). Further typing by DNA sequence analysis of the X region of the protein A gene (*spa* typing) and SCC*mec* typing were performed, as has been described, on isolates from those with clinical infections, atypical isolates from colonized horses, and a random sample of isolates from colonized horses (*12*,*26*).

Isolates from those with clinical infection were tested for the presence of the Panton-Valentine leukocidin (PVL) genes by polymerase chain reaction (PCR) and by molecular beacon with the *lukF* component of *pvl*. Amplification of the *pvl* gene was performed by using the following primers: LukS-PV: GGCCTTTCCAATACAATATTGG; and LukF-PV: CCCAATCAACTTCATAAATTG.

Thermal cycling consisted of initial heating at 95°C for 5 min followed by 35 cycles of denaturation (1 min at 94°C), annealing (30 s at 57°C), and extension (1 min at 72°C). The beacon experiment was carried out using the following beacon and primers: *lukF* beacon: 5′ 6-FAM d (CGCGAAGAATTTATTGGTGTCCTATCTCGATCGCG) DABCYL 3′, LukF F: 5′-GCCAGTGTTATCCAGAGG-3′, LukF R: CTATCCAGTTGAAGTTGATCC-3′.

Quantitative real-time PCR mixture contained 1x I.Q. supermix (Bio-Rad Laboratories Ltd., Hercules, CA, USA), 0.1 μmol/L each molecular beacon, 0.5 μmol/L of each primer, and DNA template. The thermal cycling program consisted of 10 min on a spectrofluorometric thermal cycler at 95°C, followed by 45 cycles of 30 s at 95°C, 30 s at 50°C, and 30 s at 72°C.

### Statistical Analysis

Duration of carriage by adult horses versus foals was compared by using the Mann-Whitney test. The incidence of intermittent MRSA shedding by adult horses and foals was compared by using Fisher exact test. A p value of <0.05 was considered significant for all comparisons.

## Results

In total, MRSA was isolated from 79 horses and 27 persons. Two equine isolations occurred in 2000, 5 in 2001, and 72 in 2002. Of the 79 equine cases, 27 (34%) were in horses that had been hospitalized at OVC-VTH, 41 (52%) were from 1 thoroughbred farm (farm A), and 11 (14%) were from other Ontario farms. Twenty-four of the 79 (30%) horses were adults; the remaining 55 (70%) were <1 year of age.

Clinical infections developed in 13 (16%) horses at 1 or more body sites. Incision or wound infections (n = 6), infection from intravenous catheter (n = 2), bacteremia (n = 2), pneumonia (n = 1), infection from surgical implant (n = 1), septic arthritis (n = 1), omphalophlebitis (n = 1), gluteal abscess (n = 1), and osteomyelitis (n = 1) were identified. In 2 neonatal foals, MRSA was isolated from the nares, blood, and intravenous (jugular) catheter; septic arthritis developed in 1 of the foals. The intravenous catheter was thought to be the original site of infection in both cases. Ten (85%) of 13 clinically affected horses had a history of contact with colonized persons; 2 of these also had contact with infected or colonized horses. No history of known contact with colonized humans or horses was present in 3 (15%) cases. Although many of the clinically infected horses were seriously ill and required prolonged hospitalization, MRSA was implicated directly in the death of 1 horse as a result of severe osteomyelitis.

The clinical significance of 3 isolates was unclear. One was from a routine prebreeding uterine swab from a mare without any apparent clinical abnormalities or history of reproductive disease. Two were from uterine swabs from 2 mares following abortions. Whether MRSA was the cause of abortion in either case was unclear. The remaining 63 (80%) horses had subclinical infection and were nasal carriers.

Typing was performed on all 27 OVC-VTH isolates, 37 (90%) of 41 farm A isolates, and all 11 isolates from other Ontario farms. The remaining 4 isolates, all from colonized horses, were lost before being typed. Of the 75 tested isolates, including all 13 isolates from clinical infections, 72 (96%) were identified as CMRSA 5. This strain is closely related to an international epidemic strain designated the “archaic clone” (ATCC BAA-38) (*11*). Nine different subtypes of CMRSA-5 were identified from horses. All 12 tested isolates from horses with clinical infections contained SCC*mec*IV, were *spa* type 7, and did not contain PVL genes. The 2 isolates from postabortion uterine swab specimens also contained SCC*mec*IV and were negative for PVL genes; however, 1 was *spa* type 7 and the other *spa* type 235. Five isolates from colonized horses were tested, and all also contained SCC*mec*IV and were *spa* type 7. All 3 non-CMRSA-5 isolates were obtained from farm A and were similar to, but distinguishable from, CMRSA-2, *spa* type 2 and contained *SCCmec*IV.

MRSA infection or colonization was suspected of being nosocomial in 17 (63%) of 27 OVC-VTH cases and community-acquired in 3 (11%) cases. Origin of infection was unclear in the remaining 7 (26%) cases. In these cases, the nasal swab specimen was not collected on admission; MRSA was isolated from the first sample collected >72 hours after admission, and horses were admitted from farm A during a time when numerous colonized horses were on the farm.

Sixty-eight of the equine isolates were obtained during periods of organized screening at OVC-VTH or on horse farms. The remaining 11 isolates were from clinical specimens submitted directly by primary care veterinarians to a diagnostic laboratory and are excluded from prevalence calculations. In 2000, MRSA was isolated from the nasal passages of 2 (4%) of 57 horses, including the 2 initial clinical cases. In 2002, MRSA was isolated from 25 (8%) of 320 horses at OVC-VTH and 41 (13%) of 321 horses on farm A. Of the 9 other farms evaluated after identification of an infected or colonized horse at OVC-VTH, MRSA was only identified on 1 farm, where 3 (5%) of 64 of horses were colonized. MRSA was not isolated from any of 277 horses from 8 other Ontario farms.

MRSA was isolated from 27 persons; 17 (14%) of 125 of tested OVC-VTH personnel, 8 (12%) of 67 of farm A personnel, 1 owner of a horse with an MRSA wound infection and the spouse of a colonized OVC-VTH clinician. Three human isolates were obtained in 2000, and 24 were obtained in 2002. Only 1 (4%) was from a source with clinical infection, an OVC-VTH veterinarian with a tattoo site infection. That person was infected with CMRSA-5 subtype H12, a strain that contained SCC*mec*IV, was *spa* type 7, and was PVL negative, and that strain was isolated from 2 horses that had been under that person’s care for a week before the wound infection developed. All but 1 colonized person (96%) had previous contact with 1 or more MRSA-positive horses; in 24 (89%) of 27 persons, recent contact with a horse infected with an indistinguishable subtype was documented. The colonized spouse of the colonized OVC-VTH clinician reported no contact with horses; however, isolates from both of these persons were indistinguishable from an isolate recovered from a horse under that clinician’s care. CMRSA-5 was isolated from 26 (96%) of 27 persons. One person harbored both CMRSA-5 and the CMRSA-2–like isolate in his nose at the same time. This person was a veterinarian from farm A, which was the origin of the 3 horses colonized with this strain. Nine different subtypes of CMRSA-5 were identified among human isolates.

All but 2 of the human isolates were obtained during organized screening of personnel from OVC-VTH or selected Ontario horse farms. In 2000, MRSA was isolated from 2 (10%) of 21 humans at the OVC-VTH. In 2002, MRSA was isolated from 15 (12%) of 127 persons at OVC-VTH and 8 (12%) of 68 from farm A.

Antimicrobial susceptibility testing was performed on 67 of the 72 equine and all 26 human CMRSA-5 isolates, and the 5 CMRSA-2–related strains. Five of the equine CMRSA-5 isolates were unavailable for testing. All 101 MRSA isolates tested were susceptible to ciprofloxacin, clindamycin, fusidic acid, linezolid, mupirocin, quinupristin-dalfopristin, and vancomycin. The range of oxacillin MIC was 1–>32 μg/mL, and although 21.8% of strains had oxacillin MIC of <2 μg/mL at 24 h, all such strains grew on the NCCLS oxacillin screen agar and produced the PBP2a protein. Isolates of both CMRSA-5 and CMRSA-2–related strains that were erythromycin-resistant were found to be inducibly resistant to clindamycin when challenged by using the double disk approximation test. The remaining susceptibility test results are presented in the Table.

**Table:** In vitro antimicrobial susceptibilities of 101 methicillin-resistant *Staphylococcus aureus* isolates from humans and horses*

Antimicrobial agent	MIC in μg/mL			% of isolates	
	50%	90%	Range	Intermediate	Resistant
Tetracycline	>16	>16	<4–>16	0	96
Doxycycline	8	8	<4–8	78.2	0
Minocycline	<4	<4	<4–8	14.8	0
Erythromycin	>8	>8	<0.5–>8	0	86.1
Gentamicin	>16†	>16†	<2–>16†	9.9	88.1
Rifampicin	>4	>4	<1–>4	10.9	71.2
TMP/SMX	8/152	>8/152	<2/38–8/152	NA	79.2

*MIC, minimum inhibitory concentration; TMP/SMX, trimethoprim-sulfamethoxazole; NA, no intermediate MIC category in NCCLS guidelines. †All strains were susceptible to gentamicin at a concentration of 500 μg/mL.

## Discussion

This study has identified the largest number of reported cases of clinical MRSA infection in horses and horse personnel. It also identified extensive nasal colonization in horses and horse personnel from a veterinary hospital and horse farm, nosocomial infection in a veterinary hospital setting with clinical illness in horses, and for the first time, clinical infection in 1 person working with infected horses. The subtyping information and timing of isolation provide solid evidence supporting both human-to-horse and horse-to-human transmission.

The prevalence of MRSA colonization in horses at the OVC-VTH was 4% in 2000 and 8% in 2002; however, care must be taken when interpreting these data because screening was performed during 2 periods that followed identification of clinical MRSA infection in horses at the facility. Similar limitations are present with the prevalence data regarding MRSA colonization on breeding farms, which ranged from 0% to 13% and were based on screening after identification of MRSA infection or colonization at OVC-VTH in horses from these farms. The prevalence of human colonization at OVC-VTH and 1 Ontario horse farm is of concern, particularly because of the likelihood of transmission between horses and humans on these farms. As with horses, the prevalence data in humans must be interpreted with care because of the nature of sampling. Further studies are required to determine the prevalence of MRSA infection and colonization in horses and humans at veterinary hospitals and equine farms. One well-recognized human *S. aureus* strain, CMRSA-5, a relatively uncommon isolate in Canada (*11*), has the ability to colonize the nose of horses and to spread between horses and between horses and persons on farms and within a veterinary hospital setting. The PFGE pattern of the CMRSA-5 isolates was similar to the PFGE patterns published in a previous report of MRSA infection in horses (*13*). This finding, along with the isolation of CMRSA-5 from horses in Prince Edward Island, Canada (J.S. Weese et al., unpub. data) and Colorado, USA (P. Morley, pers. comm.) that did not have any contact with colonized Ontario horses or horse personnel, further suggests that CMRSA-5 may be more disseminated in the horse population beyond Ontario.

Why MRSA has emerged in the equine population is unclear. It may reflect increased exposure of horses to MRSA-infected persons, a unique ability of CMRSA-5 to colonize horses, the increasing use of certain antimicrobial drugs in veterinary medicine, or a combination of these factors. Several recent investigations in humans suggest that the fluoroquinolones themselves may actually predispose patients to infection with or carriage of MRSA (*27*,*28*). Two case-control studies examining risk factors for MRSA found a significant association between fluoroquinolone exposure and MRSA isolation or infection (*4*,*29*).

Whether fluoroquinolone use in horses has facilitated emergence of MRSA is unclear, since no current data exist on fluoroquinolone use in veterinary medicine. Enrofloxacin is widely used in some sectors of the Ontario horse population, particularly racing horses; however, it is rarely prescribed at OVC-VTH and uncommonly used on breeding farms (J.S. Weese, unpub. data).

While MRSA has been considered primarily a hospital-associated pathogen in humans (*10*), the increasing incidence of community-acquired infection is concerning (*8*,*9*,*30*,*31*). Similar to the equine and equine-associated cases reported here, community-acquired infection with SCC*mec*IV strains has been reported in humans (*32*–*34*). Most reports of community-acquired-MRSA in humans involve skin and soft tissue infections, and production of PVL has been implicated as the possible virulence factor in community-associated MRSA infection in humans (*31*,*35*). None of the isolates from clinical equine or equine-associated infections in this study contained PVL genes; however, definitive conclusions regarding the role of PVL in equine-associated infection cannot be made with the reasonably small number of clinical isolates evaluated here. Isolates in this study were also multidrug resistant, in contrast to results of many reports of community-associated MRSA in humans (*36*,*37*). Therefore, determining the origin of these isolates (community versus hospital) is not straightforward and requires further study.

The lack of proven, safe, and acceptable options of eradication of nasal colonization in horses creates potential management problems. To date, isolation of infected horses and use of barrier precautions have been employed (J.S. Weese, unpub data). However, such methods may be difficult on farms, particularly if colonization is prolonged.

Our study has shown that MRSA infection may be an emerging disease in horses, which agrees with earlier reports (*13*,*14*). MRSA infection also may become an important nosocomial problem in the veterinary hospital setting and become endemic on horse farms, particularly in foals. Because of the extensive movement of horses, especially thoroughbreds and standardbreds, between and within Canada and the United States, MRSA colonization and infection may be more widespread than recognized. Emergence of MRSA as an equine pathogen is of additional concern because horses may be a community reservoir of MRSA and source of infection or reinfection for persons. In view of the size of the North American horse population and the frequent close contact between many persons and horses, this concern must not be dismissed. Further study is required to clarify the role of this pathogen in equine disease and transmission between horses and humans.
